# Open reduction and internal fixation using multiple nonabsorbable suture materials in acute patella fracture: comparison of clinical and radiological outcome with tension band wiring

**DOI:** 10.1186/s43019-021-00116-0

**Published:** 2021-09-28

**Authors:** Kwang Won Lee, Sang Beom Ma, Dae Suk Yang, Seung Hak Oh, Seong Ho Park

**Affiliations:** 1 Department of Orthopaedic Surgery, College of Medicine, Eulji University Hospital, 46, Dunsanseo-ro, Seo-gu, Daejeon, Republic of Korea; 2 Department of Orthopaedic Surgery, S&K Hospital, Daejeon, Korea

**Keywords:** Patella fracture, Internal fixation, Tension band wiring, Nonabsorbable suture materials

## Abstract

**Background:**

For treating displaced patella fractures, tension band wiring is the most widely used technique. However, implant removal surgery is often necessary to alleviate discomfort caused by fixation materials. On the contrary, fixation using nonabsorbable suture materials is anticipated to result in comparable outcomes without need for further implant removal surgery. However, there is a lack of clinical studies comparing the two fixation techniques (wire and nonabsorbable suture materials) for acute patella fractures.

**Methods:**

From 2014 to 2018, we retrospectively reviewed 60 patients who underwent open reduction with internal fixation for acute patella fracture. Thirty patients (group 1) who received surgery using tension band wiring and 30 patients (group 2) who received surgery using nonabsorbable suture materials were enrolled. The average follow-up period was more than 1 year after operation. Operation time, postoperative bone union time, range of motion (ROM) of the knee joint, postoperative clinical results, and complications were compared between the two groups.

**Result:**

Operation time, clinical bone union, and radiologic bone union were not statistically different between groups 1 and 2. At 3 months postoperatively, flexion was 120.3 ± 9.4° in group 1 and 110.5 ± 7.7° in group 2, showing statistically significant difference (*p* = 0.037). At 6 and 12 months postoperatively, the ROM was similar in both groups. Hospital for special surgery score at 3 months postoperatively was 78.4 ± 8.2 in group 1 and 83.7 ± 8.7 in group 2, showing statistically significant differences (*p* = 0.032). However, at 6 and 12 months postoperatively, there were no statistical differences. Lysholm score at 3 months postoperatively was 73.5 ± 8.1 in group 1 and 80.4 ± 8.2 in group 2, showing statistically significant difference (*p* = 0.016), but at 6 and 12 months postoperatively, there were no statistical differences.

**Conclusion:**

Fixation using multiple nonabsorbable suture materials can be an alternative surgical method in managing patella fractures, along with tension band wiring.

## Introduction

Acute patella fractures account for 0.8–1.5% of all fractures [1]. The most common cause of the patella fracture is a direct trauma from fall or flexion injury, and the types of fractures vary widely [2]. Since the patella acts as a fulcrum of the lever on the femoral quadriceps, playing a role in increasing the extension force of the knee joint, a functional problem of the patella causes extension complications, resulting in functional impairment in the knee joint. Thus, the purpose of the treatment of patella fracture is anatomical reduction of the articular surface and assurance of continuity in extension mechanism. Surgical treatment is generally recommended if the displacement of the fracture is greater than 3 mm, or if a mismatch in articular surface greater than 2 mm or an abnormal extension function exists [3, 4].

There are several methods for fixating patella fractures, but the most commonly used method is tension band wiring using 2k-wires and 18 gauge stainless wires for fixation. This method neutralizes traction force in anterior portion caused by extension mechanism while knee flexing, and converts it to compression force to conduct bone union in the articular surface of the patella. Most studies showed good result of tension band wiring. Therefore, it is widely used for transverse, comminuted patella fractures [4]. However, the soft tissue covering the patella is thin, the implant often causes discomfort, and it may lead to delay in wound healing, postoperative adhesion, and limitations in the knee joint range of motion (ROM) [2]. In particular, k-wires may break or loosen, causing complications such as pain, loss of reduction, displacement of fracture, and irritation of soft tissue, leading to secondary surgery for additional internal fixation and implant removal in 40% of the cases [5–7]. It is reported that, alternatively to k-wires, cannulated screws or different types of stainless wires can be used for fixation; however, up to 33% of cases still showed complications arising from the implants [2, 8]. Furthermore, in cases of severely comminuted fractures, anatomical reduction may be difficult, and an increased possibility of displacement and loosening associated with osteoporosis limits the use of tension band wiring [2]. There have been several previously published studies demonstrating fixation using high-resistance suture materials, but there was a lack of studies comparing such methods with the conventional fixation methods using metal implants [9–12].

In the present study, we assessed clinical and radiological results of fixation using nonabsorbable suture materials, compared with the conventional fixation using k-wire and tension band wiring in managing patella fractures. We hypothesized that fixation using multiple nonabsorbable suture materials would lead to less occurrence of complications and superior clinical outcomes, compared with the conventional tension band wiring method.

## Materials and methods

This study was conducted retrospectively with the approval from the institutional review board at Eulji University Hospital (EMC 2019-06-039). Surgical indications of acute patellar fracture were as follows: displacement of bone fragment greater than 3 mm, mismatch of articular surface greater than 2 mm, or extension abnormality. From 2014 to July of 2016, tension band wiring using k-wire and stainless wire was performed, and from August of 2016 to 2018, nonabsorbable suture materials (ethibond 2-0, Ethicon) were used for fixation. From 2014 to 2018, patients who underwent open reduction and internal fixation using either tension band wiring method or nonabsorbable suture materials for acute patellar fracture, with a minimum 1-year follow-up were enrolled in the study. Exclusion criteria were as follows: other surgical history of the injured site, open fractures, longitudinal fractures not requiring surgery, patella fracture accompanied with other adjacent structural injuries, and underlying significant osteoarthritis of the affected knee joint. Finally, 30 patients who underwent internal fixation using tension band wiring method (group 1) and 30 patients who underwent internal fixation using multiple nonabsorbable suture materials (group 2) were investigated in this study. We reviewed patients’ characteristics, including age, sex, follow-up duration, dominant or nondominant side, bone mineral density T-score, fracture type, and degree of displacement, and we compared the operative time, bone union period, ROM of the knee joint, postoperative functional scores, and complications.

**Fig. 1:**
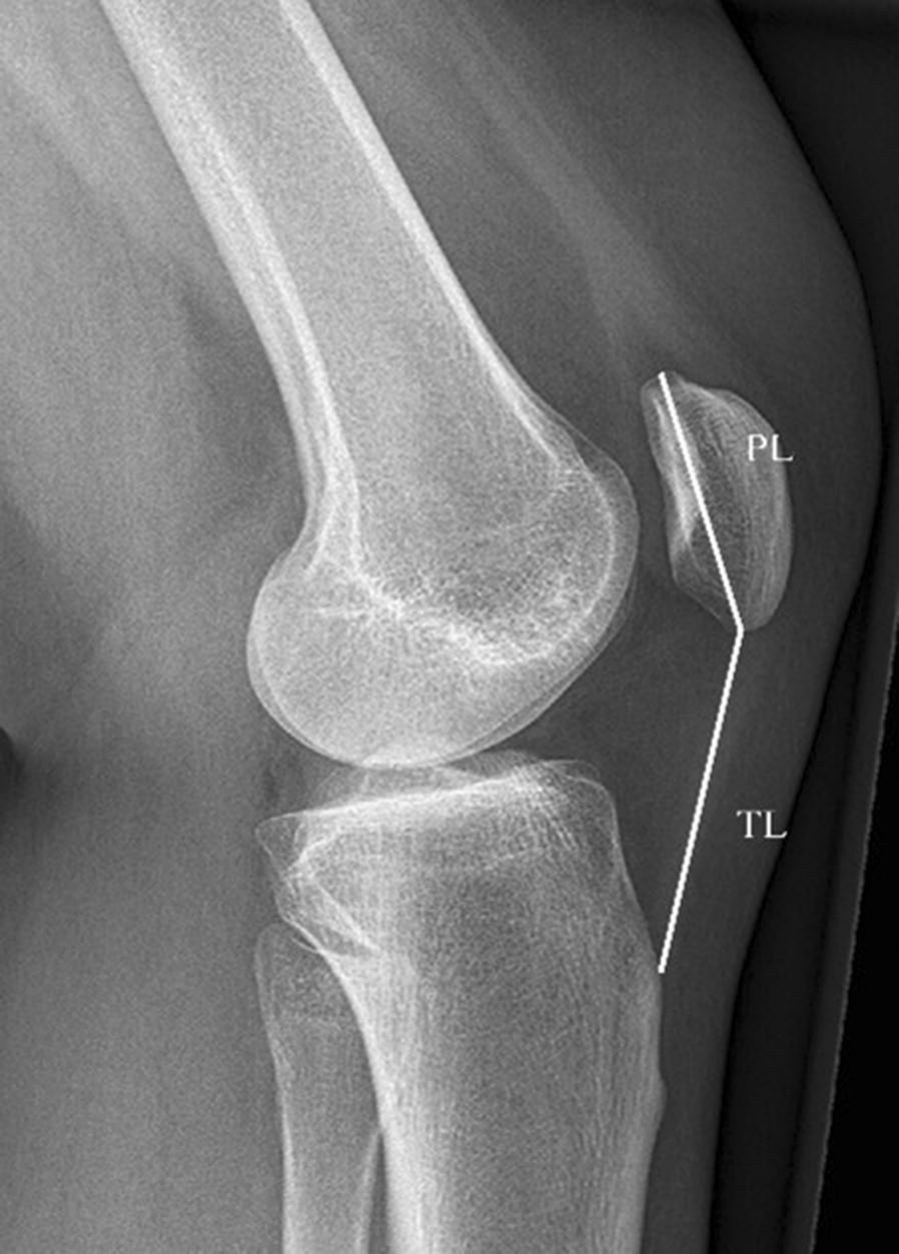
Insall–Salvati ratio. On a 30° flexed lateral knee x-ray, draw two distance lines (*PL* patella length, *TL* patellar tendon length)

For radiological evaluation, all patients were subject to simple radiographs, including anterior–posterior and lateral views of the knee joint, preoperatively and 3 months, 6 months, and 12 months postoperatively. Bone union was defined as when callus was connected between the bone fragments under simple radiograph. To evaluate the radiographic changes arising from the differences in surgical method and postoperative exercise, simple lateral radiographs of both knees were taken at 12 months postoperatively, and the length of the patella and Insall–Salvati ratio were measured and compared between the two groups (Fig. 1).

To compare postoperative clinical results, the ROM of the knee joint, Hospital for Special Surgery Score (HSS), and Lysholm score were measured at 3, 6, and 12 months postoperatively [13, 14]. Clinical bone union was defined as there being significant reduction of pain and weight-bearing ability. In addition, the postoperative complications in groups 1 and 2 were noted.

### Surgical techniques

#### Group 1

Open reduction and internal fixation procedure based on the conventional tension band wiring method was performed. After reduction of the fracture, a 1.6 mm k-wire was passed parallel in the medial and lateral 1/3 portion of the patella. Figure-of-eight tension band wiring was performed by inserting an 18 gauge wire in the medial side of the quadriceps femoris, passing through the anterior portion of the patella and inferior portion of the patella tendon. The wire knot was made in either the medial or lateral side of the superior portion of the patella. Afterwards, the k-wire was cut and bent as short as possible, and the tip was rotated, posturing so that the proximal part was placed inside the quadriceps femoris and the distal part was located inside the patella tendon to minimize skin irritation. If the comminuted fracture was difficult to fix with only the tension band, additional k-wire or cerclage wiring was applied to fix it.

#### Group 2

**Fig. 2:**
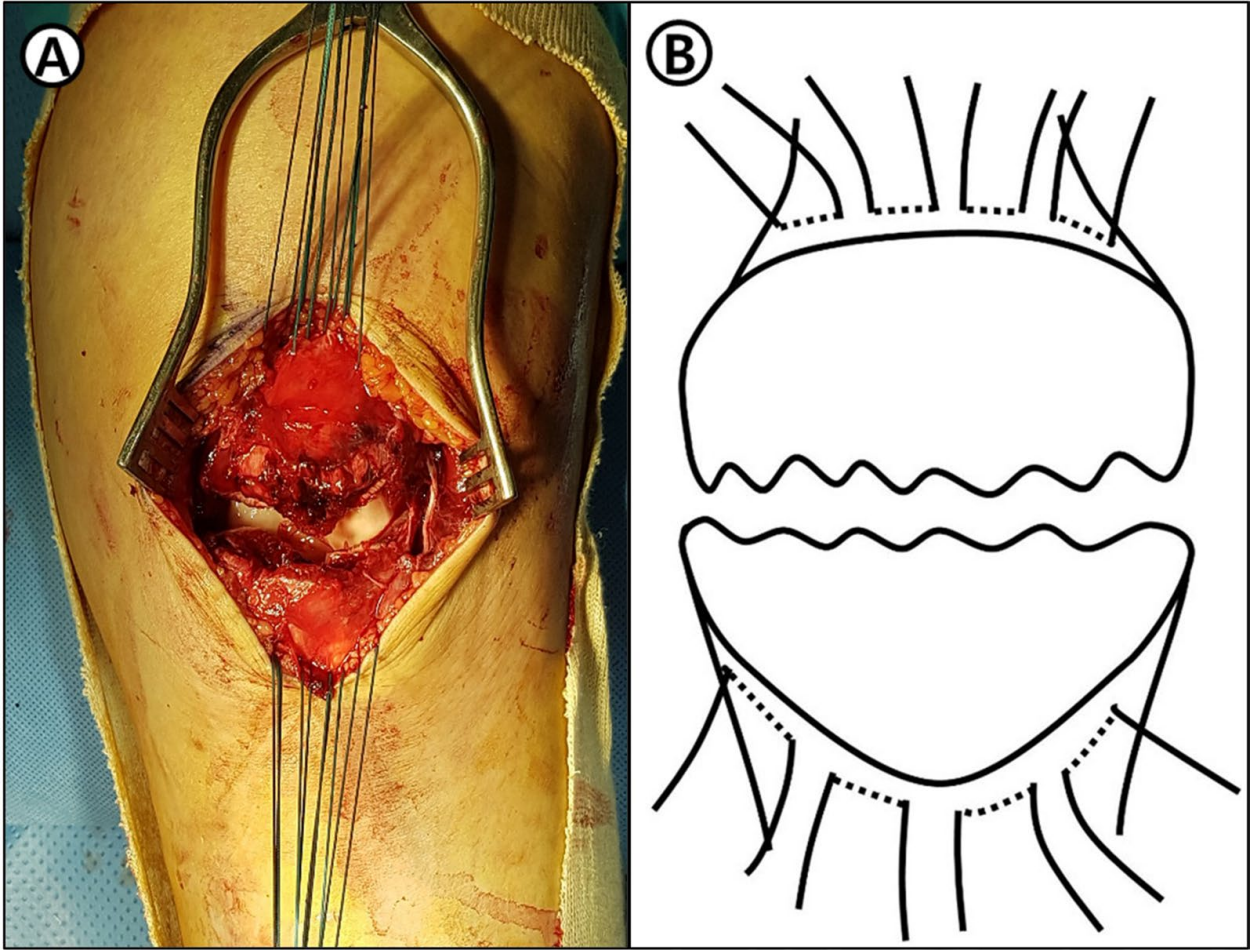
**A** Intraoperative photographs demonstrating attachment site of quadriceps femoris and patella tendon with the patella and passed through the fracture site and into the multiple layers of tendon using inside-out technique making multiple loops. **B** Scheme of this content

**Fig. 3:**
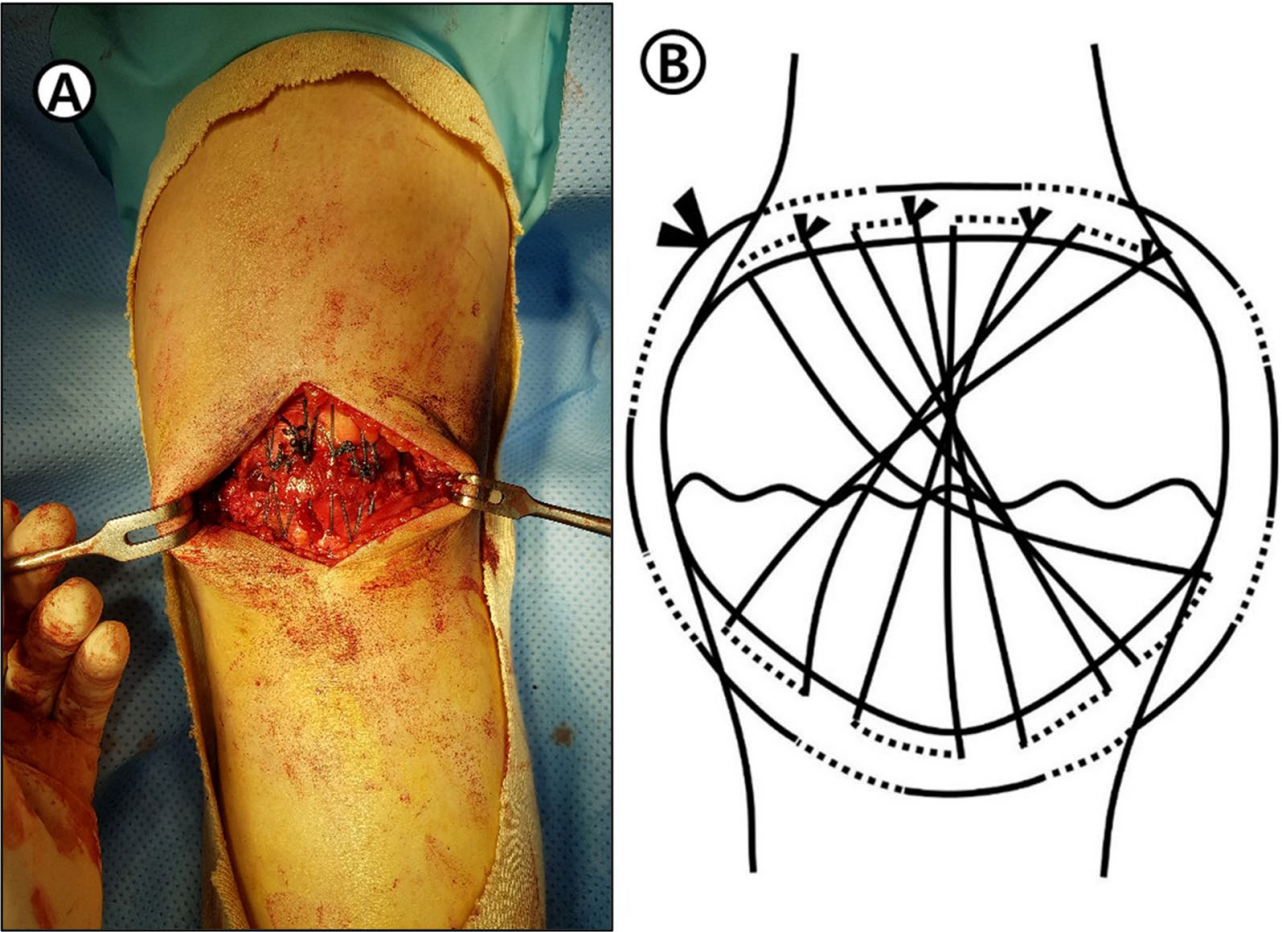
**A** Intraoperative photographs demonstrating the circumferential fixation added by passing a nonabsorbable suture through the quadriceps femoris and patella tendon. **B** Scheme of this content

Each of four to six nonabsorbable suture materials (Ethibond 2-0, Ethicon, Somervile, NJ) is inserted into the attachment site of the quadriceps femoris and patella tendon with the patella and passed through the fracture site and into the multiple layers of tendon using inside-out technique making multiple loops (Fig. 2). Afterwards, the fracture was temporarily fixed using towel clamps, and reduction was checked using C-arm fluoroscopy. If the satisfactory reduction of the fracture site and the articular surface is confirmed, the suture materials of the proximal part and the absorptive suturing of the distal part are pulled in various directions along the fracture line, and the tie was made in the best direction to maintain firm reduction and maximize fixation force. Additional tie was made to support fixation. To reduce skin irritation and discomfort after surgery, the suture knot was close to the quadriceps femoris tendon. Additionally, the circumferential fixation was added by passing nonabsorbable suture materials through the quadriceps femoris and patella tendon (Fig. 3).

If the medial and lateral retinaculum were injured, repair using absorbable suture materials was performed and passive knee ROM motion was performed to confirm the stability of the surgical fixation. Afterwards, skin suture was performed after irrigation and surgery were completed.

### Rehabilitation after surgery

#### Group 1

Partial weight bearing using two crutches, passive knee ROM, and quadriceps femoris setting exercise (QSE) were carried out 1 day after the surgery. Active ROM was started 3 weeks after surgery, and full weight bearing was performed 8 weeks after surgery.

#### Group 2

Postoperative rehabilitation was performed more slowly than in group 1. The knee joints were maintained with long leg cast in full extension for 4 weeks. From the day after surgery, partial weight bearing was performed using crutches, and QSE and straight leg raise were performed. Four weeks after surgery, the splints were removed, and full weight bearing and passive ROM were gradually applied.

### Statistical analysis

SPSS version 20.0 (IBM Corporation, Armonk, NY) was used for statistical analysis. An independent-sample Student’s *t*-test was used to compare the continuous variables including the demographics, clinical, and radiographic outcome measurements. The *χ*^2^ test was used to compare the categorical variables of patients’ demographics between the two groups. A *p*-value of < 0.05 was considered statistically significant.

## Results

The average age was 54.2 ± 10.6 in group 1 and 53.8 ± 11.6 in group 2. The average follow-up duration was 17.3 ± 4.49 months for group 1 and 16.7 ± 3.96 months for group 2. There were no significant differences in age, affected side, degree of displacement, operative time, and subtypes of fracture between the two groups (Table 1). The ratio of the patella height of the affected and the contralateral knee measured at final follow-up was 1.02 ± 0.06 in group 1 and 1.01 ± 0.05 in group 2, showing no statistical difference between the two groups (*p* = 0.154). In addition, Insall–Salvati ratio of the affected and the contralateral knee measured 0.96 ± 0.05 in group 1 and 0.99 ± 0.05 in group 2, showing no statistical difference between the two groups (*p* = 0.252) (Table 2). Clinical bone union time was 6.77 ± 0.7 weeks for group 1 and 6.21 ± 0.8 weeks for group 2 (*p* = 0.421), while radiographic bone union time was 11.3 ± 1.4 weeks for group 1 and 10.8 ± 1.5 weeks for group 2 (*p* = 0.262). These results showed no statistically significant difference between the groups.

**Table 1:** Patients demographics data

	Group 1 (*n* = 30)	Group 2 (*n* = 30)	*p*-Value
Average age (years) [range]	54.2 ± 10.6 [33–73]	53.8 ± 11.6 [36–69]	0.724
Sex (no. of patients)			
Male/female	11/19	10/20	0.787
Mean follow-up period (months)	17.3 ± 4.5	16.7 ± 4.0	0.268
Average operation time (min)	47.6 ± 7.5	45.6 ± 8.4	0.264
Dominant/nondominant side (no. of patients)	20/10	16/14	0.430
Mean BMD T-score	−1.9 ± 0.4	−1.8 ± 0.5	0.868
Subtype (no. of patients)			0.837
Transverse + comminuted	10	12	
Transverse	16	15	
Comminuted	4	3	

*BMD* bone mineral density

^*^p < 0.05 using an independent-sample Student’s *t*-test and *χ*^2^ test

**Table 2:** Clinical and radiographic outcome measurements

	Group 1	Group 2	*p*-Value
Ratio of patella height (injured/uninjured)	1.02 ± 0.06	1.01 ± 0.05	0.154
Insall–Salvati ratio (injured/uninjured)	0.96 ± 0.05	0.99 ± 0.05	0.252
Clinical bone union time (weeks)	6.77 ± 0.7	6.21 ± 0.8	0.421
Radiologic bone union time (weeks)	11.3 ± 1.4	10.8 ± 1.5	0.262
ROM			
FC (degrees) at 3 months	2.5 ± 2.2	2.3 ± 2.7	0.128
FF (degrees) at 3 months	120.3 ± 9.4	110.5 ± 7.7	0.037*
FC (degrees) at 6 months	0.8 ± 1.3	1.0 ± 1.6	0.264
FF (degrees) at 6 months	125.5 ± 10.1	124.4 ± 9.8	0.233
FC (degrees) at 12 months	0.5 ± 0.8	0.7 ± 0.9	0.436
FF (degrees) at 12 months	134.2 ± 11.2	133.6 ± 10.9	0.367
HSS score			
At 3 months	78.4 ± 8.2	83.7 ± 8.7	0.032*
At 6 months	91.3 ± 10.2	92.1 ± 10.1	0.235
At 12 months	94.2 ± 11.2	94.3 ± 10.9	0.623
Lysholm score			
At 3 months	73.5 ± 8.1	80.4 ± 8.2	0.016*
At 6 months	86.3 ± 9.7	86.9 ± 10.1	0.127
At 12 months	92.3 ± 12.3	92.6 ± 10.4	0.273

*FC* flexion contracture, *FF* forward flexion, *HSS* Hospital for Special Surgery

^*^p- < 0.05 using an independent-sample Student’s *t*-test. The values are given as the mean

### Range of motion

The values of ROM of the knee joint measured at 3, 6, and 12 months postoperatively were compared between the two groups. At 3 months postoperatively, the flexion contracture measured 2.5 ± 2.2° in group 1 and 2.3 ± 2.7° in group 2 (*p* = 0.128), showing no statistical difference. However, further flexion measured 120.3 ± 9.4 in group 1 and 110.5 ± 7.7 in group 2, showing a statistical difference between the two groups (*p* = 0.037). However, at 6 and 12 months postoperatively, there were no statistical differences of further flexion and flexion contracture between the groups.

### Clinical outcome

Both HSS and Lysholm score were evaluated at 3, 6, and 12 months postoperatively. HSS at 3 months postoperatively was measured as 78.4 ± 8.2 in group I and 83.7 ± 8.7 in group 2, with statistically significant difference between the two groups (*p* = 0.032), but there were no statistical differences between the two groups at 6 and 12 months postoperatively. Lysholm score at 3 months postoperatively was measured as 73.5 ± 8.1 in group 1 and 80.4 ± 8.2 in group 2, with statistically significant difference between the two groups (*p* = 0.016), but there were no statistical differences between the two groups at 6 and 12 months postoperatively.

### Complications

**Fig. 4:**
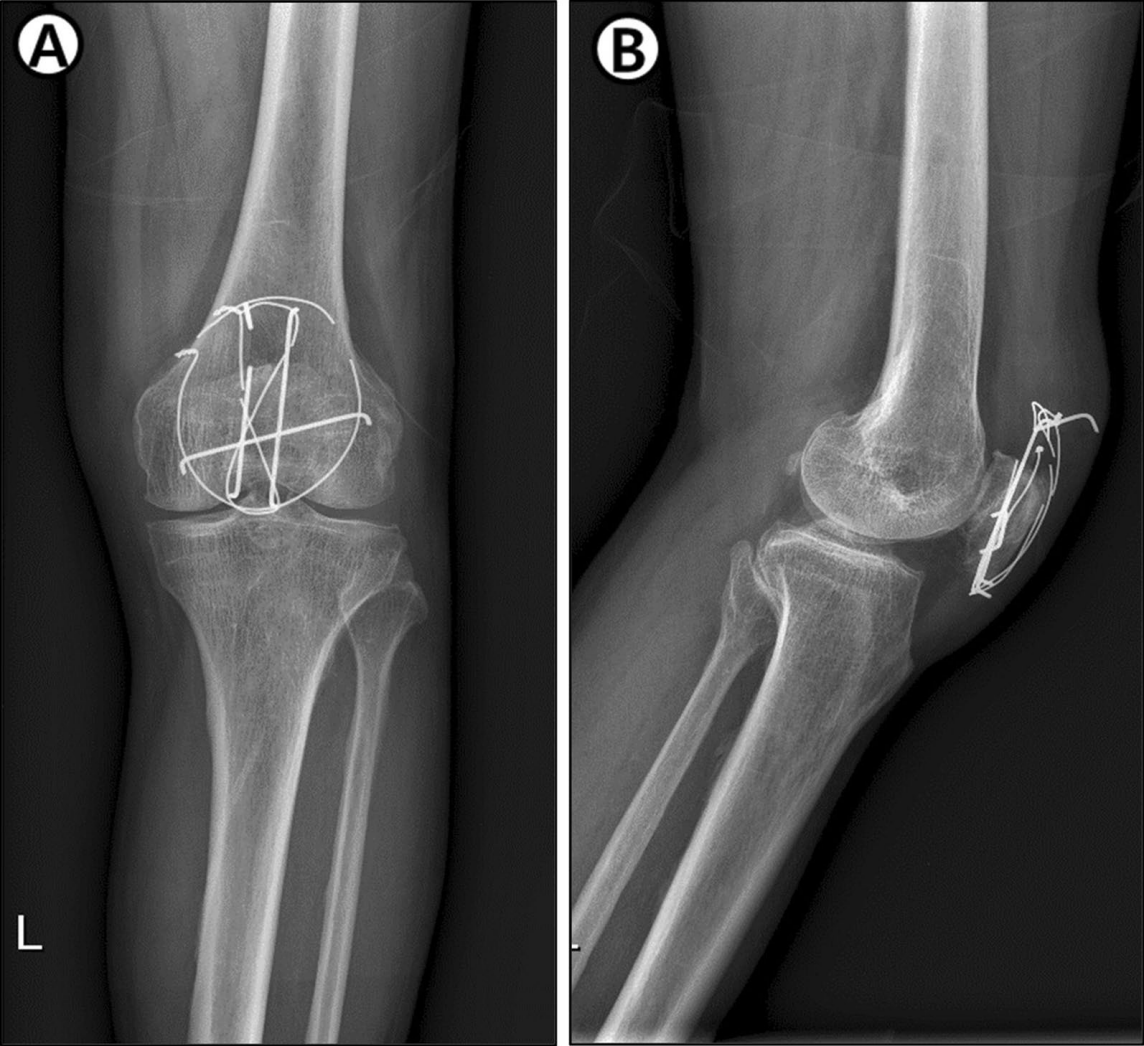
Knee anterior–posterior (AP) and lateral radiograph showing implant breakage in group 1

During the follow-up period, there were 12 cases (40%) in group 1 who wanted implant removal surgery owing to discomfort, and of those cases, 3 (10%) patients had implant breakage (Fig. 4). In group 2, there were no patients who needed implant removal. However, one case with signs of superficial infection and one case with reduction failure were identified; after cast removal at 4 weeks postoperatively, the patient started flexion exercises of the knee joint and there was loss in reduction. However, after 2 weeks of additional immobilization, the loss of reduction did not progress, and finally it was recovered without additional surgery.

## Discussion

In this study, we described the clinical and radiographic outcomes of internal fixation of acute patella fractures, comparing the two different fixation techniques (tension band wiring group and multiple nonabsorbable suture materials group). Both groups showed similar operative time and equally satisfactory bone union and radiologic outcomes. However, at 3 months postoperatively, the range of further flexion was higher in the tension band wiring group. On the contrary, functional scores (HSS and Lysholm score) were slightly better in the nonabsorbable suture materials group at 3 months postoperatively. Finally, at 6 and 12 months postoperatively, the ROM and functional scores were similar in both groups. Twelve patients (40%) of the tension band wiring group needed implant removal owing to implant discomfort or breakage, while no patient in the suture materials group needed additional surgery.

The patella plays an important role in the extension mechanism of the knee joint; thus, to reduce complications, accurate anatomical reduction, stable internal fixation, and early joint exercises are recommended in patella fractures [4]. Although internal fixation using tension band wiring is the most commonly used surgical method, it is difficult in cases of comminuted fractures or severe osteoarthritis. Reduction is especially challenging when the fracture is present in the inferior pole. The most common complication arising from internal fixation using tension band wiring is discomfort associated with the implants, often requiring secondary surgery. Kumar et al. reported surgical results and the rates of implant removal after fixation with implants in patella fractures [2]. In terms of bone union, internal fixation using metal implants is useful; however, one-third of the cases showed complications related to the implant, and 40% of the patients under 60 years of age required implant removal surgery. In addition, in patients more than 60 years of age, due to many reasons such as osteoporosis, rates of loosening and displacement of the metal implants are higher, requiring other surgical strategies to avoid secondary surgery for implant removal.

Several researchers have conducted various studies to overcome these shortcomings of tension band wiring using k-wires and stainless wires. Wright et al. conducted a biomechanical study to compare fixation using tension band wiring and suture materials in transverse patella fractures [10]. They analyzed fixation power and stiffness and concluded that fixation using suture material showed outcomes comparable to those of tension band wiring method. Camarda et al. reported good clinical results using high-tension nonabsorbable suture materials such as FiberWire in fixating patella fracture [11], and conducted a systemic review on using nonmetal implants in patella fractures [15]. The review included nine studies and 123 patients, with a 90% of success rate. Of the 123 patients, 59% had transverse fractures, 27.6% had comminuted fractures, and 13% had inferior and superior pole fractures. Although each study had different types of patients, fractures, and surgical method, only four patients (3.2%) needed additional surgery such as implant removal. This is an impressive result, compared with the reports in which 10–52% required implant removal surgery after internal fixation using metal implants. In the present study, among those who had undergone fixation using tension band wiring, 12 cases (40%) needed implant removal due to discomfort or breakage of implants. On the contrary, there were no cases that needed revision surgery or implant removal surgery among those who had undergone fixation with multiple nonabsorbable suture materials.

Clinical results such as postoperative knee joint ROM and HSS and Lysholm score of those who had undergone fixation with tension band wiring had better results, compared with those who had undergone fixation using multiple nonabsorbable suture materials at 3 months postoperatively. However, from 6 months postoperatively onward, there was no statistical difference between the methods. It is believed that the group that had undergone fixation using multiple nonabsorbable suture materials started knee joint exercises later than the group that had undergone fixation using tension band wiring because of the 4-week fixation period after surgery. After 6 months, there was no difference between the two groups.

Limitations of this study include the following: First, it was a retrospective study conducted at a single center. Secondly, since the rehabilitation process was not the same between the groups, the comparison might not be equal, affecting the outcomes. Thirdly, this study retrospectively compared the outcomes of two different surgical techniques performed at different periods (group 1 was from 2016 to 2018 and group 2 was from 2014 to 2016), without propensity score matching method. Despite similar demographic between the groups, selection bias might exist in this comparison. Finally, further studies including randomized control design with larger population and longer follow-up are anticipated to support our results.

## Conclusions

Fixation using multiple nonabsorbable suture materials showed equally satisfactory clinical and radiologic outcomes, compared with the conventional tension band wiring for acute patella fractures, and the nonabsorbable suture material group needed no additional fixation materials removal surgery. Therefore, fixation using multiple nonabsorbable suture materials can be considered as an alternative surgical method, along with fixation with tension band wiring using k-wires and stainless wires, in managing acute patella fractures.

## Data Availability

The data and materials of this study would be shared if necessary.

## References

[1] Galla M, Lobenhoffer P (2005). Patella fractures. Chirurg.

[2] Kumar G, Mereddy PK, Hakkalamani S, Donnachie NJ (2010). Implant removal following surgical stabilization of patella fracture. Orthopedics.

[3] Biddau F, Fioriti M, Benelli G (2006). Migration of a broken cerclage wire from the patella into the heart. A case report. J Bone Joint Surg Am.

[4] Carpenter JE, Kasman R, Matthews LS (1994). Fractures of the patella. Instr Course Lect.

[5] Gardner MJ, Griffith MH, Lawrence BD, Lorich DG (2005). Complete exposure of the articular surface for fixation of patellar fractures. J Orthop Trauma.

[6] Gosal HS, Singh P, Field RE (2001). Clinical experience of patellar fracture fixation using metal wire or non absorbable polyester—a study of 37 cases. Injury.

[7] Wu CC, Tai CL, Chen WJ (2001). Patellar tension band wiring: a revised technique. Arch Orthop Trauma Surg.

[8] Petrie J, Sassoon A, Langford J (2013). Complications of patellar fracture repair: treatment and results. J Knee Surg.

[9] Monaco E, Bruni G, Daggett M, Saithna A, Cardarelli S, Proietti L (2020). Patellar fracture fixation using suture tape cerclage. Arthrosc Tech.

[10] Wright PB, Kosmopoulos V, Cote RE, Tayag TJ, Nana AD (2009). FiberWire is superior in strength to stainless steel wire for tension band fixation of transverse patellar fractures. Injury.

[11] Camarda L, La Gattuta A, Butera M, Siragusa F, D'Arienzo M (2016). FiberWire tension band for patellar fractures. J Orthop Traumatol.

[12] Buezo O, Cuscó X, Seijas R, Sallent A, Ares O, Álvarez-Díaz P (2015). Patellar fractures: an innovative surgical technique with transosseous suture to avoid implant removal. Surg Innov.

[13] Baldini A, Anderson JA, Zampetti P, Pavlov H, Sculco TP (2006). A new patellofemoral scoring system for total knee arthroplasty. Clin Orthop Relat Res.

[14] Tegner Y, Lysholm J (1985). Rating systems in the evaluation of knee ligament injuries. Clin Orthop Relat Res.

[15] Camarda L, Morello S, Balistreri F, D'Arienzo A, D'Arienzo M (2016). Non-metallic implant for patellar fracture fixation: a systematic review. Injury.

